# A framework for discrete stochastic simulation on 3D moving boundary domains

**DOI:** 10.1063/1.4967338

**Published:** 2016-11-14

**Authors:** Brian Drawert, Stefan Hellander, Michael Trogdon, Tau-Mu Yi, Linda Petzold

**Affiliations:** 1) Department of Computer Science, University of California-Santa Barbara, Santa Barbara, California 93106, USA; 2) Department of Mechanical Engineering, University of California-Santa Barbara, Santa Barbara, California 93106, USA; 3) Department of Molecular, Cellular, and Developmental Biology, University of California-Santa Barbara, Santa Barbara, California 93106, USA

## Abstract

We have developed a method for modeling spatial stochastic biochemical reactions in complex, three-dimensional, and time-dependent domains using the reaction-diffusion master equation formalism. In particular, we look to address the fully coupled problems that arise in systems biology where the shape and mechanical properties of a cell are determined by the state of the biochemistry and vice versa. To validate our method and characterize the error involved, we compare our results for a carefully constructed test problem to those of a microscale implementation. We demonstrate the effectiveness of our method by simulating a model of polarization and shmoo formation during the mating of yeast. The method is generally applicable to problems in systems biology where biochemistry and mechanics are coupled, and spatial stochastic effects are critical.

## I. INTRODUCTION

Stochastic simulation of biochemical reactions has become an essential part of systems biology. Many examples exist where mean-field or deterministic analysis is insufficient to capture the relevant dynamics of real biological systems.^1–3^ In particular, systems in which the copy number of any relevant species is small will often be more accurately modeled with stochastic simulation. There exist several methods to model biochemical reactions stochastically, the most popular of which is the Stochastic Simulation Algorithm (SSA) or Gillespie algorithm.^4^ This algorithm assumes that the system is spatially homogeneous, or well-mixed, which is not the case in many interesting biological problems.

Polarization in yeast^5^ and neutrophils,^6^ Min oscillations during cell division of *E. coli*,^7,8^ and development^9^ are a few examples where the well-mixed assumption does not apply. There are several different methods for modeling spatial stochastic biochemical reactions, which can be broadly grouped into two categories. First are the particle-tracking, or free-space based on the Brownian dynamics formalism,^10^ methods that resolve the system on a microscopic scale.^11–13^ These methods are more accurate but can be quite difficult to simulate in an efficient manner. The other group of methods works on the mesoscopic scale and is based on the reaction-diffusion master equation (RDME) formalism.^7,14–16^ These methods discretize the domain into spatially homogeneous subvolumes (or voxels). Reactions within a voxel are modeled with the SSA algorithm, while diffusion between voxels is modeled as events occurring at intensities chosen to be consistent with the diffusion equation. The RDME is significantly faster to simulate than microscopic methods, with some sacrifice in accuracy. A more detailed background on these methods will be given in Section II A. One assumption that underlies all of the methods mentioned here is that the physical domain is static in time. This is often not the case in biology.

Shmoo growth during the mating of yeast,^17^ tip growth in fungal hyphae,^18^ chemotaxis in neutrophils,^6^ cell migration,^19^ and cell division^7,8^ are some examples where the physical domain of the cell is changing in time. There has also been recent work dealing with the critical role that geometry can play in fundamental biological processes, such as polarization.^20^ In this paper, we present a method to efficiently model stochastic reaction-diffusion systems in complicated, three dimensional (3D), time-dependent geometries using the RDME framework. Previous work to model stochastic reaction-diffusion systems in time-dependent domains has focused on particle-based approaches.^21^ While these methods are viable in some settings, it is generally accepted that the accurate particle-tracking methods become prohibitively expensive as the system size grows large. An efficient implementation of the RDME on time-dependent domains can effectively handle complex geometries as well as large reaction networks, while still being practical in terms of simulation time. There has also been previous work that has dealt with particle migration on 1D growing domains using the RDME formalism, where results have been compared to those of partial differential equation (PDE) models.^22–24^ Additionally, we note that the particle-based software Smoldyn^11^ has capabilities to change the size of domain during simulation.

**FIG. 1.**
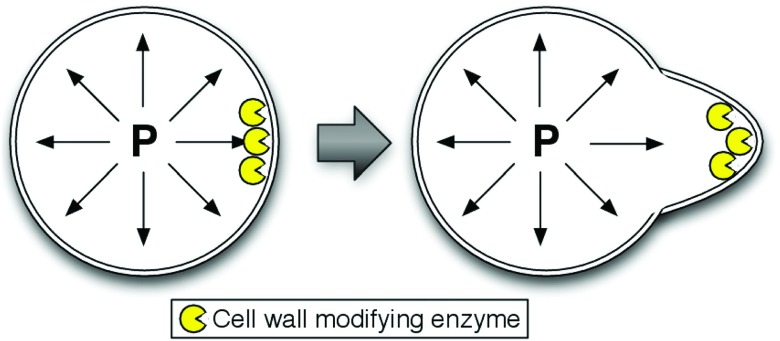
Diagram illustrating the how the yeast mating projection grows, our motivating example. Cell wall modifying enzymes (yellow) are localized to the polarized region of the cell membrane. These enzymes soften the cell wall. The internal turgor pressure pushes on the cell wall, deforming it, and creating the mating projection.

A key problem that our method addresses arises in systems where the biochemical reaction network is fully coupled to the mechanical properties of the physical domain. Our motivating example is the growth of the mating projection in yeast. In this system, enzymes modify the material properties of the cell wall, softening it, and as a result the force of the internal turgor pressure deforms the cell. At the same time, cell wall construction proteins strengthen the cell wall and slow the movement. A diagram of this process is illustrated in Figure 1. Our method does not propose to solve arbitrary mechanics of the model system under consideration. Instead, we integrate an external function or software that models and solves the equations governing the mechanics of the system, which will take as input the state of our biochemical system and provide as output the instantaneous velocity of the boundary of the domain. It is important to note, however, that this is simply one possible problem that can be handled by our proposed algorithm. In general, the function that moves the boundary does not need to be defined by mechanics (e.g., a constantly expanding sphere). The only requirement is that there is a velocity field provided to move the boundary, which can be empirically or theoretically derived.

It is important to note that in our method it is necessary to have a separation of time scales between the diffusion of the biochemical species and the movement of the boundary. It is critical that diffusion is faster than the boundary velocity to accurately simulate the system. In Sections IV A and IV B we characterize the error our method incurs and how it relates to the difference in time scales of diffusion and the velocity of the boundary. Essentially, it is possible to find a time step small enough to satisfy a user specified error tolerance if there is in fact a separation of time scales.

In this paper, we present a method to efficiently simulate stochastic reaction-diffusion models coupled to time-dependent domains using the RDME formalism. An outline for the rest of this paper is as follows: in Section II, we give a brief review of the RDME and how it can be simulated efficiently in complex domains using the Next Subvolume Method (NSM)^15^ on unstructured meshes.^25^ In Section III, we present our method and discuss implementation and theoretical considerations. Next, in Section IV, we present three examples that serve to show the convergence properties and accuracy of the method along with demonstrating the applicability of the method to biologically relevant problems. Specifically, in Section IV A, we compare the results of our method to a microscale implementation of a single species diffusing within an expanding 1D line that can react with the boundary. This example serves to demonstrate the accuracy and convergence (in the spatial distribution of molecules) of our method compared with a microscale implementation over a range of expansion velocities and time steps. To accurately compare these different scales of simulation, we also derived the relationship between mesoscale and microscale reaction rates (see Appendix C for details). Next, in Section IV B, we present a more biologically relevant, yet still theoretically tractable, model of polarization in yeast, introduced in Ref. 26. This model contains a density dependent switch for polarization which we explore through an expanding and contracting sphere and compare to theoretical steady state results from Ref. 26. Lastly, in Section IV C we present a model in which the state of the biochemical system dictates the movement of the boundary. In particular, we present a new model for the polarization of Cdc42 in mating yeast (see Appendix Appendix A for details) and qualitatively compare to experimental data. Finally, we end the paper in Section V with a discussion of the method and our results.

## II. BACKGROUND

### A. The reaction-diffusion master equation

As mentioned above, the RDME^27^ is a mesoscopic model for spatial stochastic chemical reactions. It gives the time evolution of the probability distribution for the state of the system. First, the physical domain is partitioned into *K* nonoverlapping subvolumes or voxels, similar to numerical methods for PDEs. Molecules are taken to be point particles and the state of the system is the discrete number of molecules of each species for each of the voxels in the mesh. The computational mesh can either be a structured Cartesian grid or an unstructured triangular or tetrahedral mesh. Here, we focus on the unstructured case which will be discussed further in Section II C. Modeling the reaction-diffusion dynamics as a Markov process gives the following forward Kolmogorov equation for the time evolution of *p*(x,*t*) = *p*(*x*,*t*|*x*_0_,*t*_0_) (the probability that the system can be found in state x at time *t*, conditioned on the initial condition x_0_ at time *t*_0_)

$$\frac{\partial p\left(\text{𝐱},t\right)}{\partial t}=ℛp\left(\text{𝐱},t\right)+𝒟p\left(\text{𝐱},t\right),$$ (1)

$$ℛp\left(\text{𝐱},t\right)=\sum_{i=1}^{K}\sum_{r=1}^{M}a_{ir}\left(\text{𝐱}-\nu_{ir}\right)p\left(\text{𝐱}-\nu_{ir},t\right)-a_{ir}\left(\text{𝐱}\right)p\left(\text{𝐱},t\right),$$ (2)

$$𝒟p\left(\text{𝐱},t\right)=\sum_{s=1}^{N}\sum_{i=1}^{K}\sum_{j=1}^{K}d_{sij}\left(\text{𝐱}-\mu_{sij}\right)p\left(\text{𝐱}-\mu_{sij},t\right)-d_{sij}\left(\text{𝐱}\right)p\left(\text{𝐱},t\right),$$ (3)

where ${\text{𝐱}}_{i\cdot }$ denotes the *i*th row and ${\text{𝐱}}_{\cdot j}$ denotes the *j*th column of the K×S state matrix *x*, where *S* is the number of chemical species. The functions *a*_*ir*_(*x*_*i*_) define the propensity functions of the *M* chemical reactions, and $\nu_{ir}$ are stoichiometry vectors associated with the reactions. The propensity functions are defined such that $a_{ir}\left(\text{𝐱}\right)\Delta t$ gives the probability that reaction *r* occurs in a small time interval of length Δt. The stoichiometry vector $\nu_{ir}$ defines the rules for how the state changes when reaction *r* is executed. *d*_*ijk*_(*x*_*i*_) are propensities for the diffusion jump events, and $\mu_{ijk}$ are stoichiometry vectors for diffusion events. $\mu_{ijk}$ has only two non-zero entries, corresponding to the removal of one molecule of species *x*_*k*_ in voxel *i* and the addition of a molecule in voxel *j*. The propensity functions for the diffusion jumps, *d*_*ijk*_, are selected to provide a consistent and local discretization of the diffusion equation, or equivalently the Fokker-Planck equation for Brownian motion. It is important to note, as mentioned in Section I, that this formalism is defined for a physical domain that is static in time. We will relax this assumption with our method to accommodate time-dependent domains.

### B. The next subvolume method

In most cases, the RDME is too high-dimensional to solve directly. Thus, algorithms have been developed that generate exact realizations of the Markov process described by the RDME, in a Monte Carlo fashion. One particularly efficient algorithm that we focus on for our implementation is the Next Subvolume Method (NSM).^15^ In this algorithm, the time to the next event in each voxel (either a chemical reaction or diffusion event) calculated by the Direct Method formulation of the SSA.^28^ To identify in which voxel the event occurs, the algorithm uses the Next Reaction Method formulation of the SSA.^29^ If it was a chemical reaction event that occurred, then only the voxel in which the event occurred needs to be updated, while if a diffusion event occurs both the voxel where the molecule started and the voxel where the molecule ended up need to be updated. The key to the efficiency of the NSM is the use of an event priority queue which gives a scaling of $𝒪\left(\log_{2}\left(K\right)\right)$, where *K* is the number of voxels in the mesh.

### C. RDME on unstructured meshes

The use of unstructured meshes allows for complicated geometries in 3D to be more easily accommodated, such as the curved surfaces of cell membranes. PyURDME^30^ (based on URDME^25^) is a software framework for simulation of the RDME on unstructured meshes that we extend to time-dependent domains. For the theoretical details of how to obtain mesoscopic diffusion constants on unstructured meshes, see Ref. 31. Using the finite element package DOLFIN^32^ we obtain the diffusion matrix for the system, from which we get the jump coefficients for individual voxels. The flexibility of simulating on unstructured meshes allows our method to handle complex time-dependent domains in 3D.

## III. COMPUTATIONAL METHOD FOR SPATIAL STOCHASTIC SIMULATION WITH A MOVING BOUNDARY

In this section we develop a computational method for simulation of spatial stochastic systems defined by the RDME formalism on domains with moving boundaries. Our method utilizes the time scale separation between the diffusion of the biochemical species in the RDME system and the movement of the boundary of the domain. The method is formulated for systems where diffusion is faster than the boundary movement. In this context we use operator splitting to decouple the reaction-diffusion operator from the domain movement operator, solving each operator sequentially over the same time step.

In the formulation of the RDME, the spatial domain is discretized into volume units known as voxels. For example, in our PyURDME software package^30^ the 3D domain is discretized using tetrahedral elements. Since the RDME is formulated to be solved on a static domain, we cannot directly adapt it to a moving domain. Instead, we employ an operator splitting style simulation, by first solving the RDME for a small time τ (the splitting time step) on a static mesh. Then we use the state of the biochemistry to find the velocity of the boundary of the domain through a function specified by the user, and evolve the mesh over the same time step τ. Finally, the state of the biochemical system is transformed to the newly evolved mesh. A sequence of these steps are taken until the simulation reaches the final time.

**FIG. 2.**
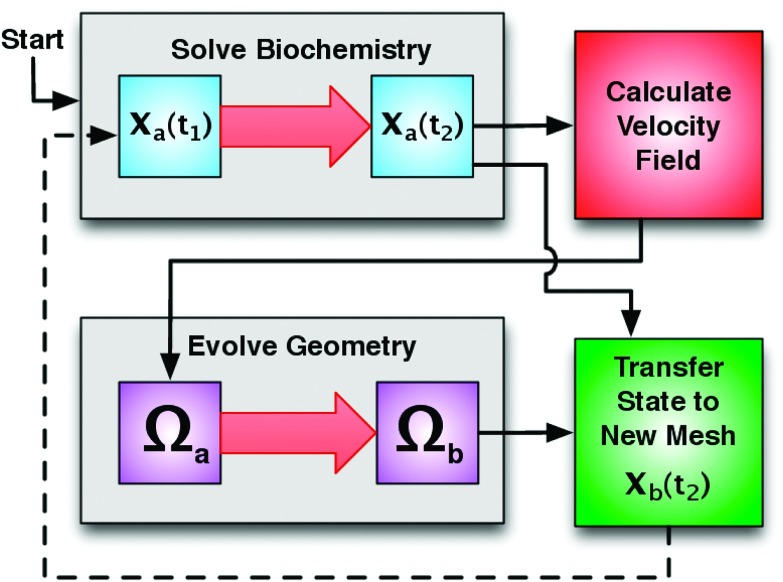
Diagram illustrating the process flow of the moving mesh algorithm.

The algorithm for moving the mesh has four components. Figure 2 illustrates these components and the process flow between them. The first component is the simulation of the biochemical system for a time τ on a specific mesh (denoted as $\Omega_{a}$) starting from an initial state *x*_*a*_(*t*) to a final state ${\text{𝐱}}_{a}\left(t+\tau \right)$,

$${\text{𝐱}}_{a}\left(t+\tau \right)=\text{RDME}\left({\text{𝐱}}_{a}\left(t\right),\Omega_{a},\tau \right).$$ (4)

The second component is the computation of the velocity field *v* at the boundary of the domain, as a function the state of the biochemical system. In our motivating example of the growth of the yeast mating projection, the internal turgor pressure pushes uniformly on the cell wall, but the wall expands preferentially where it has been softened by the enzymes. Thus, the instantaneous velocity field of the growing mating projection is a function of the spatial distribution of the cell wall modifying enzymes within cell. See Section IV C for more details, and Figure 7 for an illustration.

This is the component that couples the biochemical simulation with the moving domain. This function is a part of the model being simulated, and is thus provided by the user as input to the method. The function can be of only the final state of the biochemical system, ${\text{𝐱}}_{a}\left(t+\tau \right)$, or of some aggregate of all previous states, ${\text{𝐱}}_{a}\left(0:t+\tau \right)$,

$$v=\frac{d\Omega }{dt}{|}_{t+\tau }=f_{\Delta \Omega }\left({\text{𝐱}}_{a}\left(0:t+\tau \right)\right).$$ (5)

The third component is the evolution of the mesh over the simulation time step τ. Using the domain boundary velocity calculated previously, a new mesh is created by evolving the mesh linearly over the time step. This is done by moving each mesh point according to the velocity field at that point, via the forward Euler method. In our software implementation, this is done via the FEniCS/Dolfin package,^32^

$$\Omega_{b}=\Omega_{a}+\tau v.$$ (6)

**FIG. 3.**
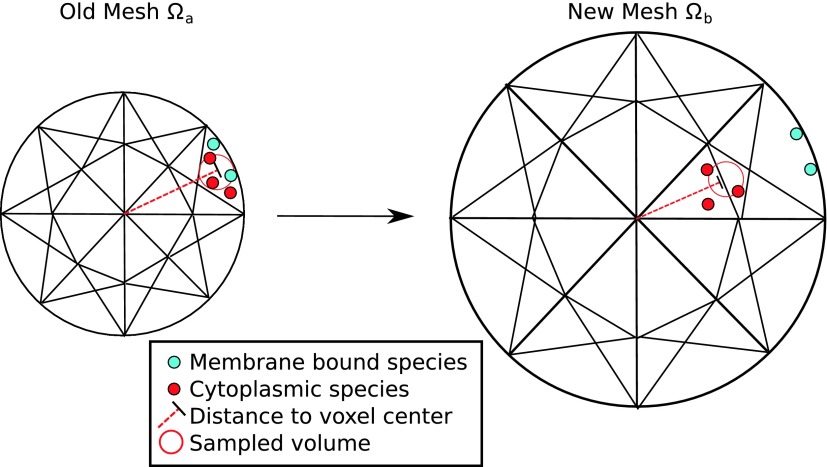
Diagram illustrating the particle redistribution process. On each step of the moving mesh simulation, the state of the biochemical system is transferred from the old mesh (left) to the new mesh (right). The *x*, *y*, *z* position of each particle is sampled on the old mesh (uniformly from within the volume of the containing voxel); the particle is assigned to the voxel in the new mesh that is closest to that sampled position. If the species of a particle is restricted to a subdomain (e.g., membrane-bound proteins), then it is moved to the closest voxel in that subdomain.

The final component of the algorithm is the method for transferring the state of the biochemical system from the current mesh, $\Omega_{a}$, to the newly created mesh, $\Omega_{b}$. On each step of the moving mesh simulation the *x*, *y*, *z* position of each particle is sampled on $\Omega_{a}$. An assumption of the RDME is that particles are uniformly distributed within each voxel. Consequently, the position of the particle is sampled uniformly from its containing voxel’s volume. Since the boundaries of a voxel are often difficult to compute on an unstructured mesh, we make an approximation and sample the position from a sphere with a volume equivalent to that of the containing voxel. It is important to note that this assumption induces a spatial error that is proportional to the mesh resolution and the quality of the mesh. That is, the more elongated the tetrahedrons are, the more error is induced in the sampled particle’s spatial position. Implementations of this algorithm must ensure that the mesh is of sufficient quality throughout the simulation. Next, the particle is assigned to the closest voxel new mesh, $\Omega_{b}$ (minimizing Euclidian distance), to the sampled *x*, *y*, *z* position. Often in systems biology models, biochemical species are required to remain in specific subdomains of the system. For example, membrane bound proteins must remain on the membrane, which is modeled as the voxels on the boundary of the mesh. If the species of a particle is restricted to a subdomain in this way, then it is moved to the closest voxel that is within that subdomain. If the sampled position of a particle falls outside the domain $\Omega_{b}$, then it is placed at the closest voxel (that is of an appropriate subdomain) within $\Omega_{b}$. See Figure 3 for an illustration. This procedure is repeated for each particle within the system, thus the biochemical state of the system is transferred from $\Omega_{a}$ to $\Omega_{b}$, which we denote as

$${\text{𝐱}}_{b}\left(t\right)=\text{ParticleRedistribution}\left({\text{𝐱}}_{a}\left(t\right),\Omega_{a},\Omega_{b}\right).$$ (7)

The iterative algorithm is described in Algorithm 1.

**Table:** ALGORITHM I. Spatial stochastic simulation for a domain with a moving boundary.

**Input**: $\Omega_{0}$, $f_{\Delta \Omega }$, $\tau_{split}$, *x*_0_(0), *t*_*final*_, and the Biochemical Reaction Network
**Output**: $\left[\Omega_{0}\cdots \Omega_{n}\right]$, $\left[{\text{𝐱}}_{0}\cdots {\text{𝐱}}_{n}\right]$
1: *i* = 0, t=0, $\tau =\tau_{split}$
2: **while** t < *t*_*final*_ **do**
3: ${\text{𝐱}}_{i}\left(t:t+\tau \right)=\text{RDME}\left({\text{𝐱}}_{i}\left(t\right),\Omega_{i},\tau \right)$
4: $v=f_{\Delta \Omega }\left({\text{𝐱}}_{i}\left(t:t+\tau \right)\right)$
5: $\Omega_{i+1}=v\tau +\Omega_{i}$
6: ${\text{𝐱}}_{i+1}\left(t+\tau \right)=\text{ParticleRedistribution}\left({\text{𝐱}}_{i}\left(t+\tau \right),\Omega_{i},\Omega_{i+1}\right)$
7: *i* = *i*+1, t=t+τ
8: **end while**

### A. Rejection-based step size selection

We extend the method presented in Algorithm 1 to include an adaptive method for error control. We will see in Example 2 that the error our method incurs will depend on variables of the velocity of the domain and the operator splitting time step. To implement our adaptive error control time stepping scheme, we define a new input *d*_*max*_ as the maximum distance any given point on the boundary is allowed to move in any single time step. In each step, if the magnitude of any component of the velocity multiplied by the time step τ is greater than *d*_*max*_, then that step is rejected, the time step is set to half its previous value, and the state is recomputed over the new time step. The accepted time step will then be used for the next step of the algorithm. Finally, if the step is accepted on the first pass (no rejection) and the time step had been previously reduced (τ is less than $\tau_{split}$), then time step for the subsequent step, $\tau_{next}$, is increased to double the current time step size. See Algorithm 2 for details. Note that, as the simulation of the RDME by the NSM algorithm is a continuous operator, we are able to sample at any specified time within the interval [t,t+τ]. This allows us to avoid recomputing the biochemical state when a step is rejected, leading to a more efficient implementation. In our simulations, for each invocation of the NSM operator, we sample the state of the RDME at [$t+\frac{\tau }{8}$, $t+\frac{\tau }{4}$, $t+\frac{\tau }{2}$, t+τ]. This allows us to half the time step three times without

**Table:** ALGORITHM II. Adaptive spatial stochastic simulation for a moving boundary domain.

**Input**: $\Omega_{0}$, $f_{\Delta \Omega }$, *d*_*max*_, $\tau_{split}$, *X*_0_(0), *t*_*final*_, and the Biochemical Reaction Network
**Output**: $\left[\Omega_{0}\cdots \Omega_{n}\right]$, $\left[{\text{𝐱}}_{0}\cdots {\text{𝐱}}_{n}\right]$
1: *i* = 0, t=0, $\tau =\tau_{split}$
2: **while** t < *t*_*final*_ **do**
3: ${\text{𝐱}}_{i}\left(t:t+\tau \right)=\text{RDME}\left({\text{𝐱}}_{i}\left(t\right),\Omega_{i},\tau \right)$
4: $v=f_{\Delta \Omega }\left({\text{𝐱}}_{i}\left(t:t+\tau \right)\right)$
5: $d={||v||}_{\infty }\tau$
6: **If** $d>d_{max}$**then**
7: **repeat**
8: τ=τ/2
9 $v=f_{\Delta \Omega }\left({\text{𝐱}}_{i}\left(t:t+\tau \right)\right)$
10 $d={||v||}_{\infty }\tau$
11 $\tau_{next}$ = τ
12 **Until**$d\leq d_{max}$
13 **else if** $\tau <\tau_{split}$**then**
14 $\tau_{next}$ = 2τ
15 **end if**
16 $\Omega_{i+1}=v\tau +\Omega_{i}$
17 ${\text{𝐱}}_{i+1}\left(t+\tau \right)=\text{ParticleRedistribution}\left({\text{𝐱}}_{i}\left(t+\tau \right),\Omega_{i},\Omega_{i+1}\right)$
18 *i* = *i*+1, t=t+τ, $\tau =\tau_{next}$
19 **end while**

recomputation of the biochemical system. It should be noted that to avoid biased simulations, the state of the random number generator must be preserved when the step is rejected and the step restarted with the same state.

## IV. RESULTS

Here we present three examples to verify and show the utility of our method.

### A. Example 1

In our first example, we demonstrate numerically that our method converges in distribution as the time step decreases. In a general problem the error depends on multiple factors, such as the time step, the mesh resolution, and the quality of the mesh. To isolate the error induced by the time step selection, we consider a 1D domain Ω of width R−L, where *R* is the right endpoint and *L* the left endpoint. A single species *S* diffuses (with *D* = 1), associates with, and dissociates from the left boundary. We let Ω expand to the left; thus *L* is a function of time *t*. Specifically we let

L(t)=−vt,

where *v* is the constant speed of the expansion.

The domain is discretized into *N*_vox_ voxels, each of width *h*. We denote the microscopic association rate by *k*_*r*_ and the microscopic dissociation rate by *k*_*d*_. The mesoscopic rates, $k_{a}^{\text{meso}}$ and $k_{d}^{\text{meso}}$, are then given by $k_{a}^{\text{meso}}=k_{a}/h$ and $k_{d}^{\text{meso}}=k_{d}$, as shown in Appendix C. We let *L* = 0 and *R* = 1 initially.

To show that our method is accurate we simulate the system until the final time *T* = 1, and compare the spatial distribution of particles to the spatial distribution obtained with a more detailed Brownian dynamics method.^10^ The error will be a function of the speed of expansion *v*, the time step $\Delta t_{\text{split}}$, the number of voxels *N*_vox_, and the reaction rates *k*_*r*_ and *k*_*d*_. We expect the error to be larger for a large *v*, as the boundary moves more during each time step. For small enough *N*_vox_ the spatial resolution will be insufficient, and the error will consequently be large. To demonstrate these effects, we ran simulations with $\Delta t_{\text{split}}$ varying from 0.01 to 0.2 with $N_{\text{vox}}\in \left\{5,20,50\right\}$.

**FIG. 4.**
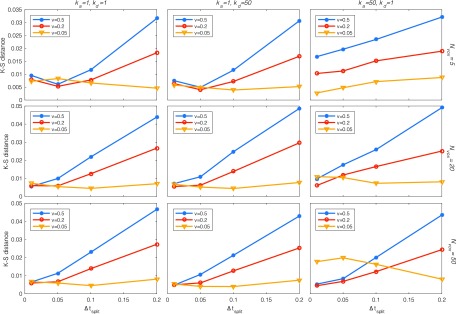
Error in distribution as a function of the size of the time step for a 1D moving domain with an absorbing/desorbing boundary for three different reactive rates (columns) and three different spatial discretizations (rows). Our mesoscopic method was compared with a Brownian dynamics microscopic simulation of $10^{6}$ molecules. We define the error as the Kolmogorov-Smirnov distance between the spatial distributions of unbound particles at the end time (1s). As we can see by comparing the middle three panels to the bottom three panels, the error is similar to *N*_*vox*_ = 20 and *N*_*vox*_ = 50, meaning that the problem is spatially well-resolved already with *N*_*vox*_ = 20, while for *N*_*vox*_ = 5 we can see that the system is not fully resolved for the case of *k*_*a*_ = 50 and *k*_*d*_ = 1.0. As expected, the larger the speed *v* of the expansion, the larger the error, but as the splitting time step $\Delta t_{\text{split}}$ decreases, so does the error. For *v* = 0.05, the domain is expanding so slowly that the stochastic error dominates, and no difference is seen as $\Delta t_{\text{split}}$ varies between 0.01 and 0.2.

We simulated 10^6^ molecules and computed the Kolmogorov-Smirnov distance between the spatial distributions of unbound particles at the final time point *T* (with the domain expanding at constant velocity, convergence at the final time point implies convergence throughout). In Figure 4 we show that, as expected, the error decreases with decreasing time step $\Delta t_{\text{split}}$.

### B. Example 2: Density dependent switch for polarization in an expanding and contracting sphere

In our second example, we verify the accuracy of our method by comparing against the analytical solution of a biochemical model found in the literature. To demonstrate the applicability of our method to biologically relevant problems, we have implemented a simple model of polarization in yeast on a moving domain. In particular, we focus on a model of polarization presented in Ref. 26 that relies on a minimal positive feedback circuit. This model is particularly interesting as it has been shown to polarize only when modeled stochastically, opposed to deterministically. The yeast cell is modeled as a sphere with a membrane on the surface of the sphere. The model has three reactions between two species: cytosolic Cdc42 spontaneously attaches to the membrane with rate *k*_*on*_ (Eq. (8)), membrane-bound Cdc42 likewise spontaneously detaches with rate *k*_*off*_ (Eq. (9)), and finally membrane-bound Cdc42 recruits cytosolic Cdc42 to the membrane at rate *k*_*fb*_, to close the positive feedback loop (Eq. (10)),

$$Cdc42_{c}\overset{k_{on}}{\to }Cdc42_{m},$$ (8)

$$Cdc42_{m}\overset{k_{off}}{\to }Cdc42_{c},$$ (9)

$$Cdc42_{c}+Cdc42_{m}\overset{k_{fb}}{\to }2Cdc42_{m}.$$ (10)

The cytosolic and membrane-bound species can diffuse at rates *D*_*cyt*_ and *D*_*mem*_, respectively, (the diffusion of the membrane-bound Cdc42 being restricted to the membrane). This model was shown in Ref. 26 to have a density dependent switch. That is, there is a critical range for polarization of molecules on the membrane. This range is from a lower critical density necessary to facilitate polarization to an upper density above which molecules become essentially homogeneous on the membrane (i.e., not polarized). From Ref. 26 it is also possible to calculate theoretically the steady state ratio of molecules in the cytoplasm for any given density, which we will use as a comparison for our simulations.

**FIG. 5.**
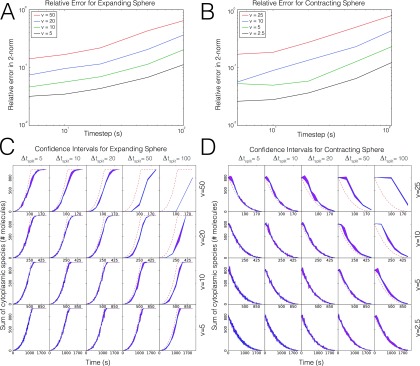
Comparison of our method to theoretical results from the literature. As expected, the error for our method decreases with the velocity of the boundary and the time step. (a) Relative error in 2-norm for a variety of expansion velocities (v) and operator-split time steps for an expanding sphere. (b) Relative error in 2-norm for a variety of contraction velocities (v) and operator-split time steps for a contracting sphere. (c) 95% confidence intervals for three trajectories at a variety of expansion velocities (v) and operator-split time steps (Δt_split_) for an expanding sphere, along with the theoretical value (dashed red line). (d) 95% confidence intervals for three trajectories at a variety of contraction velocities (v) and operator-split time steps (Δt_split_) for a contracting sphere, along with the theoretical value (dashed red line).

**FIG. 6.**
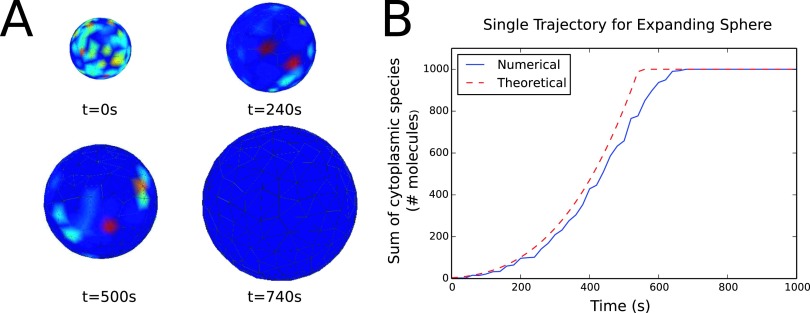
(a) Time series of heatmaps showing the number of membrane-bound molecules on the surface of an expanding sphere (note that the radii are not to scale and by 740 s there are no molecules on the membrane). (b) An example of a single trajectory showing the number of cytoplasmic molecules versus time for an expanding sphere along with the theoretical value as calculated from Ref. 26. Note that for each parameter value multiple realizations were run and averaged before being compared to the theoretical value.

To test our moving mesh algorithm, we implemented the model described above in an expanding and contracting sphere for a fixed number of total molecules. For the expanding sphere case, the initial radius was set below the theoretical switch value calculated from Ref. 26. Specifically, the critical radius can be calculated as follows:

$$\frac{4}{3}\pi r_{crit}^{3}=N\frac{k_{fb}}{k_{off}},$$ (11)

where *N* is the total number of molecules (cytosolic and membrane-bound) and *r*_*crit*_ is the critical radius. Here we set *N* = 1000 and $\frac{k_{fb}}{k_{off}}=0.9$, thus from Eq. 11 we have $r_{crit}=6.425\mu m$. From the initial radius (below the critical radius), the radius of the sphere expands at a constant velocity to a final value which is greater than the critical radius. The error incurred by our operator split method is dependent on both the speed at which the sphere expands and the operator split time step that is chosen. Again, the error here is defined as the relative error between the number of cytosolic molecules in the simulation and the theoretical steady state value calculated from Ref. 26. We calculated this error over a range of expansion velocities and operator split time steps to investigate the convergence behavior for our method. A similar test was performed for a contracting sphere which starts at a radius just below the critical value and decreases at a constant velocity to some final radius. The results of these convergence studies are shown in Fig. 5. As expected, the larger the velocity of radial expansion, the more error the method will generate for both the expanding (Fig. 5(a)) and contracting (Fig. 5(b)) sphere cases. The same trend can be seen for large time steps (Figs. 5(a) and 5(b)). Note that for each parameter value multiple realizations were run and averaged before being compared to the theoretical value. The 95% confidence interval for three realizations of the number of cytosolic molecules at each parameter value is shown for the expanding sphere in Fig. 5(c) and for the contracting sphere in Fig. 5(d). An example time series of heatmaps showing the number of membrane bound molecules on the surface of an expanding sphere can be seen in Fig. 6(a), with red being a higher number of molecules and blue a lower value. Also, a sample trajectory of the expanding sphere simulation can be seen in comparison to the theoretical steady state solution in Fig. 6(b) (here with parameter values of $\frac{dr}{dt}=10$ nm/s and *t*_*split*_ = 20 s). Again, the purpose of this example problem is to characterize the error incurred by our method as compared to theoretical results in the literature and

specifically to elucidate how this error depends on the velocity of the boundary and the time step. This example also demonstrates the utility of our method for studying problems of biological relevance.

### C. Example 3: Formation of the yeast mating projection

In our third example, we show the use of our method on a biologically relevant system that couples the biochemical reactions with the moving boundary. Our motivating example is the polarization of proteins during mating of *Saccharomyces cerevisiae*, and the resulting growth of the mating projection.

Yeast cells sense mating pheromone in their extra-cellular environment, and determine the direction of their mating partner by sensing the chemical gradient. The chemical gradient of pheromone induces polarization of the yeast cell, localizing proteins, and actin cables to the region of the yeast cell that is closest to a nearby mating partner. The mating projection starts to form when the polarisome organelle is formed at the site of polarization. The polarisome acts to coordinate the formation of the mating projection via the transport of cell wall cutting enzymes as well as cell wall material and synthase proteins. As these processes work together, the yeast cell changes shape from a spheroid to grow a projection.

**FIG. 7.**
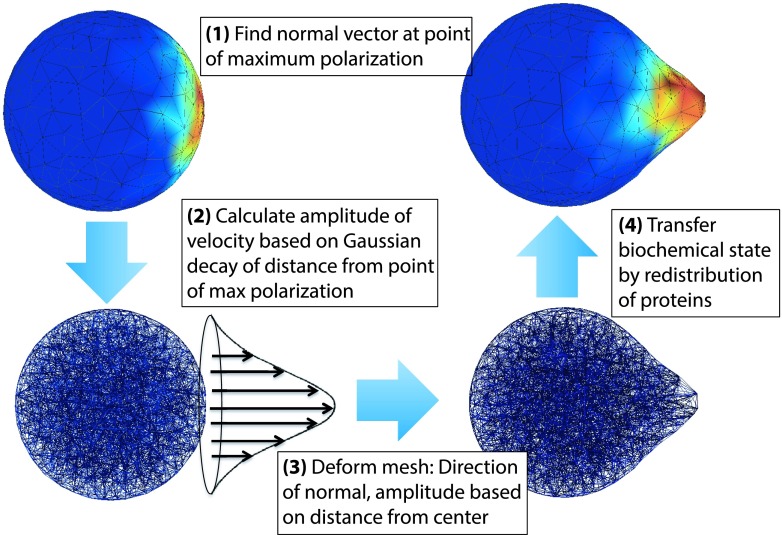
Diagram describing the process where polarization of the yeast model (spatial psrofile of protein concentration) is used to calculate the deformation of the domain. At each time step of the algorithm the following process is repeated. First the biochemical system is simulated using the spatial stochastic solvers in PyURDME for a length of Δt. Next the point of maximum polarization is found and the normal vector is calculated at that point. Then, the velocity of the surface is calculated using the normal vector as the direction, with the amplitude calculated from a Gaussian function centered at the maximum polarization point (empirically parameterized). The mesh is then deformed by the application of the velocity field. Finally, the biochemical state of the system is transferred to the new mesh.

To simulate this process we present a new spatial stochastic biochemical model of yeast polarization, centered around the polarization of the protein Cdc42. This model is a novel combination of reactions published in Refs. 33 and 34. As these models were originally presented deterministically, for use with this method we have converted the reactions to a mechanistic and stochastic formulation (see Appendix A for a complete description of the model). To present a simplified model of polarization, we omit the dynamics of the receptor-ligand binding (presented elsewhere^35,36^) and take as input to the model a time-constant spatially varying concentration of the activated G-protein (beta-gamma subunit). The biochemistry determines the moving boundary by expanding the sphere at the point of greatest polarization, in the direction of the normal at that location. The other points on the boundary of the domain are moved in a parallel direction, with the magnitude attenuated by a Gaussian of the distance to the point of maximum polarization. This is illustrated in Figure 7.

**FIG. 8.**
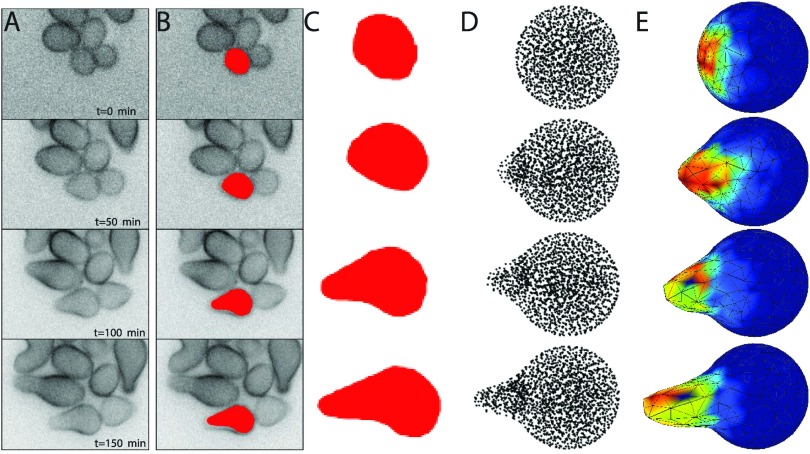
Comparison between microscopy images of yeast cells during polarized growth and simulations of the growing yeast mating projection. (a) Fluorescent microscopy time-lapse images of yeast cells during exposure to mating pheromone (*α*-factor). Cells are tagged with Mid2-GFP (see Appendix D for experimental details). (b) Manual cell shape extraction overlaid on microscopy images for a single cell. (c) Enlarged cell shape without microscopy image. (d) Scatter plot of the voxel centers of our simulation results projected onto a plane containing the origin and the point of greatest polarization. (e) 3D visualization of the simulated growing yeast cell, where the color map shows the concentration of active Cdc42 on the membrane (red corresponds to the highest concentration, and blue to the least).

Figure 8 shows a comparison between our yeast polarization simulation results (columns (d) and (e)) and microscopy images of a polarizing yeast cell (columns (a)-(c)). Column (b) shows the outline of the yeast cells overlaid on the microscopy images, and column (c) shows just the outline of these cells. Column (d) shows a scatter plot of the voxel centers of our simulation results, projected onto a plane containing the origin and the point of greatest polarization. Column (e) shows the 3D visualization of the simulated growing yeast cell, where the color map shows the concentration of active Cdc42 on the membrane (red corresponds to the highest concentration, and blue to the least). The frames (rows) in column (a)-(c) are separated by 50 min intervals. In this simulation, the state of the biochemical system and the movement of the boundary are fully coupled. This figure shows a qualitative match between a real cell phenotype and our biochemical model simulated via our method.

## V. CONCLUSIONS

We have developed a method for simulating stochastic biochemical reactions on time-dependent domains using the RDME formalism. This method involves the following steps: simulating the RDME on a fixed geometry for a given time step, using the state of the biochemical system as input to a function that moves the boundary in a user-specified manner, over that same time step, redistributing the molecules in the system to the new geometry, and then repeating until a specified end time. Our method simulates the RDME on unstructured meshes, which allow it to easily handle the complex geometries that often show up in biological applications. We have shown, through various example problems, that the error our method will incur depends on a few key factors, including the specified time step, the speed of the moving domain, the diffusion constants of the species, and the reaction rates of the system. We have also demonstrated the potential usefulness of such a method by simulating the biologically relevant problem of shmoo formation during the mating of yeast, a problem where spatial stochastic effects are important and the geometry is changing in time as a result of the state of the biochemical system.

We have implemented this method in our spatial stochastic modeling and simulation software package PyURDME:^30^ the Python package for simulation of Unstructured mesh Reaction-Diffusion Master Equation models. The reaction-diffusion biochemical model system has been extended to allow the inclusion of a movement of the mesh, and the inspection of the resulting mesh quality and adaptive mesh refinement are accomplished via integration of the open source finite element package FEniCS/Dolfin.^32^ The software package, along with instructive examples, is available from our code repositories on Github.

This method is generally applicable to problems arising in systems biology where spatial and stochastic effects are critical and the physical geometry is changing in time. In particular, this method is applicable to the common case in systems biology where the movement of the boundary of a cell is directly determined by the state of certain biochemical species. The error will be more manageable for systems where there is some separation of time scales between the movement of the boundary and the diffusion rate of the biochemical system. In the future, we hope to extend our analysis of biologically relevant problems with coupled biochemistry and domain movement, such as in shmoo formation in yeast mating. Other specific systems where this method could be useful include the following: tip growth in fungal hyphae,^18^ chemotaxis in neutrophils,^6^ cell migration,^19^ and cell division.^7,8^ Another future direction is to develop a method that more closely couples the dynamics of the moving boundary and the biochemistry, thus avoiding the error involved in splitting the two, but this is a considerably more involved problem.

## APPENDIX A: MODEL OF Cdc42 POLARIZATION IN YEAST

The model for the of *Cdc*42 used in Section IV C is a combination of reactions published in Refs. 33 and 34. The key component from Ref. 33 is what is known as a GDI reaction which preferentially moves the inactive form of *Cdc*42 from the membrane to the cytosol. Diffusion is much faster in the cytosol than on the membrane and this difference in diffusion is what leads to a Turing-type mechanism for pattern formation, as originally discussed in Ref. 37. The essential components from Ref. 34 are a negative feedback mechanism through the protein *Cla*4 and recruitment of *Cdc*24 to the membrane by *Gbg* (the βγ subunit of the heterotrimeric G-protein which is activated in response to pheremone during yeast mating). Both Refs. 33 and 34 originally modeled their respective reactions using deterministic PDE models. Here we take certain key reactions from both models and model them mechanistically using the RDME formalism, which results in the following reactions:

$$Cdc24_{c}+Gbg_{m}\overset{k_{on}}{\to }Cdc24_{m}+Gbg_{m},$$

$$Cdc24_{m}+Cdc42GDP_{m}\overset{\alpha_{1}}{\to }Cdc24_{m}+Cdc42GTP_{m},$$

$$Cdc42GTP_{m}\overset{\alpha_{3}}{\to }Cdc42GDP_{m},$$

$$Cdc24_{m}+Cdc42GDP_{c}\overset{\beta_{1}}{\to }Cdc24_{m}+Cdc42GTP_{m},$$

$$Cdc42GDP_{c}\overset{\beta_{2}}{\to }Cdc42GDP_{m},$$

$$Cdc42GDP_{m}\overset{\beta_{3}}{\to }Cdc42GDP_{c},$$

$$Bem1_{c}+Cdc42GTP_{m},$$

$$\overset{\gamma_{1}}{\to }Bem1_{m}+Cdc42GTP_{m},$$

$$Bem1_{m}\overset{\gamma_{2}}{\to }Bem1_{c}$$

$$Bem1_{m}+Cdc24_{c}\overset{\delta_{1}}{\to }Bem1_{m}+Cdc24_{m},$$

$$Cdc24_{m}\overset{\delta_{2}}{\to }Cdc24_{c},$$

$$Cdc42GTP_{m}+Cla4_{m}\overset{k_{Cla4a}}{\to }Cdc42GTP_{m}+Cla4_{am},$$

$$Cdc24_{m}+Cla4_{am}\overset{k_{24d}}{\to }Cdc24_{c}+Cla4_{am},$$

$$Cla4_{am}\overset{k_{Cla4d}}{\to }Cla4_{m}$$

where the subscripts *c* and *m* denote cytoplasm and membrane-bound species, respectively. *Cdc*42*GTP* and *Cdc*42*GDP* are the active and inactive forms of *Cdc*42 on the membrane respectively. *Cla*4_*am*_ and *Cla*4_*m*_ are the active and inactive forms of *Cla*4 on the membrane respectively. The physical domain starts as a sphere with a radius of 2.0μm with a membrane on the surface of the sphere and evolves according to the state of the biochemical system. Diffusion of membrane bound species is restricted to the membrane. *Gbg* is taken to be a constant input function with a uniform concentration throughout the domain (i.e., it is not an explicit species and thus does not diffuse). The parameter values, descriptions, and references used in Section IV C are given in Table I.

**TABLE I.** Parameters for the Cdc42 yeast polarization model.

Parameter	Value	Description	Reference
*D*_*m*_	0.0053 $\mu {\text{m}}^{2}{\text{s}}^{-1}$	Diffusion constant on	38
		membrane	
*D*_*c*_	50 $\mu {\text{m}}^{2}{\text{s}}^{-1}$	Diffusion constant in	39
		cytoplasm	
*R*	2.00 μm	Reference radius of cell	34 and 40
*N*_42_	3000	Total number of *Cdc*42	34 and 40
		molecules	
*N*_*B*_	3000	Total number of *Bem*1	34 and 40
		molecules	
*N*_24_	1000	Total number of *Cdc*24	34 and 40
		molecules	
*N*_*Cla*4_	5000	Total number of *Cla*4	34 and 40
		molecules	
*N*_*Gbg*_	5000	Total number of *Gbg*	34 and 40
		molecules	
$\alpha_{1}$	0.2 $\mu {\text{m}}^{2}{\text{s}}^{-1}$	*Cdc*24 activating *Cdc*42_*m*_	33
$\alpha_{3}$	1 $s^{-1}$	Deactivation of *Cdc*42	33
$\beta_{1}$	0.266 $\mu {\text{m}}^{3}{\text{s}}^{-1}$	*Cdc*24 activating *Cdc*42_*c*_	33
$\beta_{2}$	0.28 $\mu \text{m}{\text{s}}^{-1}$	Attachment of *Cdc*42_*c*_	33
$\beta_{3}$	1 $s^{-1}$	Detachment of *Cdc*42_*m*_	33
$\gamma_{1}$	0.2667 $\mu {\text{m}}^{3}{\text{s}}^{-1}$	Active *Cdc*42 recruit *Bem*1_*c*_	33
$\gamma_{2}$	0.35 $s^{-1}$	Detachment of *Bem*1_*m*_	33
$\delta_{1}$	0.00297 $\mu {\text{m}}^{3}{\text{s}}^{-1}$	*Bem*1_*m*_ recruit *Cdc*24_*c*_	33
$\delta_{2}$	0.35 $s^{-1}$	Detachment of *Cdc*24_*m*_	33
*k*_*on*_	0.00297 $\mu {\text{m}}^{3}{\text{s}}^{-1}$	Gbg recruit *Cdc*24_*c*_	33
*k*_*Cla*4*a*_	0.006 $s^{-1}$	Active *Cdc*42 recruit *Cla*4	34 and 40
*k*_*Cla*4*d*_	0.01 $s^{-1}$	Negative feedback via *Cla*4	34 and 40
*k*_24*d*_	1.0/30 000 $s^{-1}$	Deactivation of *Cla*4	34 and 40

## APPENDIX B: MICROSCALE SIMULATIONS

On the microscopic scale we track the continuous position of individual particles, in contrast to the mesoscopic scale on which we track only the copy number of particles in each voxel. Molecules move by normal diffusion and are modeled by hard spheres with reaction radius σ. The reaction dynamics as two molecules collide is governed by the Smoluchowski equation^41^ with a Robin boundary condition.

Consider two molecules, *S*_1_ and *S*_2_, with diffusion rates *D*_1_ and *D*_2_ and reaction radii $\sigma_{1}$ and $\sigma_{2}$. Let *D* = *D*_1_ + *D*_2_ and $\sigma =\sigma_{1}+\sigma_{2}$. At time *t*_*n*_, the positions are given by ${\text{𝐱}}_{1}^{n}$ and ${\text{𝐱}}_{2}^{n}$. We let *p*(*x*_1_, *x*_2_, *t*|*x*_1*n*_, *x*_2*n*_, *t*_*n*_) be the probability distribution function representing the probability that the positions of the molecules are given by *x*_1_ and *x*_2_ at time *t*. The diffusion of the molecules is governed by the Smoluchowski equation

$$\partial_{t}p=D_{1}\Delta_{{\text{𝐱}}_{1}}p\left({\text{𝐱}}_{1},{\text{𝐱}}_{2},t\right)+D_{2}\Delta_{{\text{𝐱}}_{2}}p\left({\text{𝐱}}_{1},{\text{𝐱}}_{2},t\right),$$ (B1)

where $\Delta_{{\text{𝐱}}_{i}}$ is the Δ-operator applied to **x**_*i*_, and where *p*(*x*_1_, *x*_2_, *t*) := *p*(*x*_1_, *x*_2_, *t*|*x*_1*n*_, *x*_2*n*_, *t*_*n*_).

Via a change of variables, $Y=X_{1}-X_{2}$ and $\text{𝐘}=\sqrt{D_{2}/D_{1}}{\text{𝐱}}_{1}+\sqrt{D_{1}/D_{2}}{\text{𝐱}}_{2}$, we obtain two independent equations

$$\partial_{t}p\left(\text{𝐘},t\right)=D\Delta_{\text{𝐘}}p\left(\text{𝐘},t\right),$$ (B2)

$$\partial_{t}p\left(\text{𝐲},t\right)=D\Delta_{\text{𝐲}}p\left(\text{𝐲},t\right).$$ (B3)

The reaction dynamics are now governed by the boundary conditions, given by

$$K\frac{\partial p_{\text{𝐲}}}{\partial n}{|}_{∥\text{𝐲}∥=\sigma }=k_{r}p_{\text{𝐲}}\left(∥\text{𝐲}∥=\sigma ,t\right),$$ (B4)

where

$$K=\left\{ \begin{array}{c} 2\pi \sigma D,\;\left(2D\right),\\ 4\pi \sigma^{2}D,\;\left(3D\right), \end{array}\right.$$ (B5)

and where *k*_*r*_ is the microscopic reaction rate.

The initial condition is given by $p\left(\text{𝐲},0|{\text{𝐲}}_{0},0\right)=\delta \left(\text{𝐲}-{\text{𝐲}}_{0}\right)$, and we enforce p(∥𝐲∥→∞)=0. New positions for two interacting molecules can be sampled by solving the above equations. An efficient algorithm for simulating systems of molecules is the GFRD algorithm.^13^ Another approach, aimed at simplifying the sampling process by solving the above equation by performing operator splitting, is described in Ref. 42. For microscopic simulations on moving meshes, we follow the approach described in Ref. 21.

## APPENDIX C: MESOSCOPIC REACTION RATES IN 1D

In Section IV A we compare mesoscopic simulations in 1D with microscopic Smoluchowski simulations in 1D. To do an accurate comparison, we first need to determine how the mesoscopic reaction rates relate to the microscopic rates. To obtain this relationship we follow an approach similar to that in Refs. 43–45.

We consider a particle of species *A* diffusing with rate *D* in a 1D domain, and reacting with the left boundary according to

$$A\overset{k_{\text{on}}}{\underset{k_{\text{off}}}{⇄}}A_{\text{mem}},$$ (C1)

where *A*_*mem*_ is a molecule of species *A* bound to the membrane. The rate *k*_*on*_ is the effective rate, by which we mean that, given a uniformly distributed *A* molecule, the molecule will react with the boundary after an average of *V*/*k*_*on*_ s, where *V* is the volume of the domain (in this case the length of the interval).

Let $k_{r}^{\text{meso}}$ (*s**−1*)be the intensity with which an *A* molecule reacts with the membrane, given that it is occupying the voxel adjacent to the membrane. First we find $k_{r}^{\text{meso}}$ as a function of *k*_*on*_ and the width *h* of the voxel, and then proceed to determine *k*_*on*_ as a function of the microscopic reaction rate *k*_*r*_.

We first note that a problem equivalent to the above is that of a molecule *A* starting uniformly on a 1D domain of width 2*V* reacting with a molecule *B* fixed at the origin (at rate *k*_*on*_). This can be seen by considering the symmetry of the problem. We therefore let *L* = 2*V*, and derive $k_{r}^{\text{meso}}\left(k_{\text{on}},h\right)$ to match the average binding time of an *A* molecule to a molecule fixed at the origin on a 1D interval of width *L*.

We denote the mean effective binding time of the two molecules by $\tau_{\text{eff}}$. Thus $\tau_{\text{eff}}=L/k_{\text{on}}$, and

$$\tau_{\text{eff}}=\tau_{\text{meso}}\left(k_{r}^{\text{meso}}\to \infty \right)+\tau_{\text{meso}}^{\text{rebind}}\left(k_{r}^{\text{meso}}\right),$$ (C2)

where $\tau_{\text{meso}}\left(k_{r}^{\text{meso}}\right)$ is, given a uniform inital distribution, the average time until the *A* molecule reacts with the *B* molecule on the mesoscopic (RDME) scale, and $\tau_{\text{meso}}^{\text{rebind}}\left(k_{r}^{\text{meso}}\right)$ is the average time until the *A* molecule rebinds to the *B* molecule, given that the molecules have just dissociated. Note that the rebinding time is the same as the time until the *A* molecule reacts with the *B* molecule, given that they start in the same voxel. For simplicity we will henceforth denote $\tau_{\text{meso}}\left(k_{r}^{\text{meso}}\to \infty \right)$ by $\tau_{\text{meso}}\left(\infty \right)$. Let *d*_tot_ denote the mesoscopic jump rate out of a voxel (so that *d*_tot_ = *h*^2^/(2*D*)). Then

$$\theta =\frac{k_{r}^{\text{meso}}}{k_{r}^{\text{meso}}+d_{\text{tot}}}$$ (C3)

is the probability that the next event, given that the *A* molecule occupies the origin voxel, is a reaction event rather than a diffusion event. Let $\tau_{e}={\left(k_{r}^{\text{meso}}+d_{\text{tot}}\right)}^{-1}$ be the average time until this event fires. We obtain

$$\tau_{\text{meso}}^{\text{rebind}}\left(k_{r}^{\text{meso}}\right)=\frac{1}{\theta }\left\{\theta \tau_{e}+\left(1-\theta \right)\left(\tau_{e}+t_{1}\right)\right\},$$ (C4)

where *t*_1_ is the average time until the *A* molecule binds to the *B* molecule, given that the molecule occupied the origin voxel and then diffused instead of reacted. Some straightforward algebra yields

$$\tau_{\text{meso}}^{\text{rebind}}\left(k_{r}^{\text{meso}}\right)=\frac{1}{k_{r}^{\text{meso}}}\left(1+d_{\text{tot}}t_{1}\right).$$ (C5)

We now use (C5) in (C2) and solve for $k_{r}^{\text{meso}}$ to obtain

$$k_{r}^{\text{meso}}=\frac{1+d_{\text{tot}}t_{1}}{\tau_{\text{eff}}-\tau_{\text{meso}}\left(\infty \right)}.$$ (C6)

In the expression above we have two unknowns: *t*_1_ and $\tau_{\text{meso}}\left(\infty \right)$. The effective binding time is a constant that we choose ($\tau_{\text{eff}}=V/k_{\text{on}}$).

From Refs. 46 and 47 we have analytical expressions for *t*_1_ and $\tau_{\text{meso}}\left(\infty \right)$,

$$1+d_{\text{tot}}t_{1}=N,$$ (C7)

$$\frac{D}{2h^{2}}\tau_{\text{meso}}\left(\infty \right)=\frac{N\left(N+1\right)}{6},$$ (C8)

where *N* is the number of voxels. We obtain

$$\tau_{\text{meso}}\left(\infty \right)=\frac{2h^{2}}{D}\frac{N\left(N+1\right)}{6}=\frac{2L^{2}+Lh}{6D},$$ (C9)

$$k_{r}^{\text{meso}}=\frac{1}{h}\frac{L}{\tau_{\text{eff}}-\frac{2L^{2}+Lh}{6D}}.$$ (C10)

Note that $\tau_{\text{eff}}$ is often considered a constant that we choose arbitrarily or obtain from experiments. However, in this case we want to compare mesoscopic simulations with microscopic simulations, and we derive $\tau_{\text{eff}}$ as a function of the microscopic rate *k*_*r*_.

The effective binding time $\tau_{\text{eff}}$ can be written in terms of microscale quantities as the sum of the time until the *A* molecule reaches the boundary for the first time, *L*^2^/(3*D*), and the time until it reacts with the boundary given that the *A* molecule starts in contact with the boundary, *L*/*k*_*r*_. We thus obtain

$$\tau_{\text{eff}}=\frac{L^{2}}{3D}+\frac{L}{k_{r}},$$ (C11)

in agreement with Ref. 48. Inserting (C11) into (C10), and assuming L≫h (so that $2L^{2}\gg Lh$), we now obtain

$$k_{r}^{\text{meso}}\approx \frac{k_{r}}{h}.$$ (C12)

Thus, when comparing a mesoscale simulation to a microscale (Smoluchowski) simulation in 1D, the mesoscopic reaction rate constant should be related to the microscopic rate according to (C12). Note that from (C12) follows that the mesoscopic dissociation rate will be given by $k_{d}^{\text{meso}}=k_{d}$ to ensure that the steady states agree.

## APPENDIX D: EXPERIMENTAL DETAILS

Time-lapse imaging was performed on Mid2-GFP cells adhered to glass slides using concanavalin A in standard yeast-peptone-dextrose (YPD) media with the stage heated to 30 °C. Both fluorescent and bright-field images were taken at 10 min intervals over a 3 h period using a Nikon TE-2000 inverted microscope with an ORCA-2 CCD camera controlled by MetaMorph software. The yeast strain was constructed by fusing GFP onto the C-terminal end of MID2 using genomic integration of a W303-1A strain that is $\bar{1}\Delta$ (RJD863).
